# Tracking the evolution of a single composite particle during redox cycling for application in H_2_ production

**DOI:** 10.1038/s41598-020-62237-y

**Published:** 2020-03-24

**Authors:** Dragos Neagu, Evangelos I. Papaioannou, Bernhard Tjaden, Xuekun Lu, Cheuk-Man Mak, Michael W. Gaultois, Brian Ray, Paul Shearing, Ian S. Metcalfe

**Affiliations:** 1 School of Engineering, Newcastle University, Merz Court, Newcastle upon Tyne, NE1 7RU UK; 2 Electrochemical Innovation Lab, Department of Chemical Engineering, University College London, London, WC1E 7JE UK; 3 Leverhulme Research Centre for Functional Materials Design, The Materials Innovation Factory, Department of Chemistry, University of Liverpool, 51 Oxford Street, Liverpool, L7 3NY UK

**Keywords:** Chemical engineering, Energy, Materials for energy and catalysis

## Abstract

Composite materials consisting of metal and metal oxide phases are being researched intensively for various energy conversion applications where they are often expected to operate under redox conditions at elevated temperature. Understanding of the dynamics of composite evolution during redox cycling is still very limited, yet critical to maximising performance and increasing durability. Here we track the microstructural evolution of a single composite particle over 200 redox cycles for hydrogen production by chemical looping, using multi-length scale X-ray computed tomography. We show that redox cycling triggers a centrifugal redispersion of the metal phase and a centripetal clustering of porosity, both seemingly driven by the asymmetric nature of oxygen exchange in composites. Initially, the particle develops a large amount of internal porosity which boosts activity, but on the long term this facilitates structural and compositional reorganisation and eventually degradation. These results provide valuable insight into redox-driven microstructural changes and also for the design of new composite materials with enhanced durability.

## Introduction

Composite materials such as metal—metal-oxide systems are often used in applications where complex functionality is required but cannot be easily met by a single-phase material. As such, composite materials are being increasingly investigated to tackle some of the challenges facing energy conversion and storage technologies including solid oxide fuel cells, thermochemical solar to fuels and chemical looping processes^1–9^. In these applications, composites are generally required to undergo repeated exposure to reducing and oxidising environments at elevated temperatures, which often leads to material and performance degradation over time. Consequently, there is considerable interest in understanding the structural and compositional changes of composites under redox conditions. This would enable better control over their evolution, performance and long-term durability, which represent some of the key challenges to address in the above-mentioned energy conversion technologies.

Chemical looping, one of the above-mentioned fields of application, has received increased attention as an alternative method for hydrogen production as compared to the conventional, energy-intensive process of steam methane reforming followed by shift and separation^8–10^. In chemical looping, redox reactions are divided into sub-reactions which are then carried out separately. Thus, the streams of reducing and oxidizing reactants never come in direct contact but exchange oxygen in separate steps, sequentially and cyclically, through a so-called oxygen carrier material (OCM). One critical parameter used to characterize the effectiveness of OCMs is their oxygen capacity (δ), i.e. the extent to which they can reversibly release/incorporate oxygen under redox conditions. This can be expressed on a mass basis (δ_m_), or on a molar basis (δ_M_), and is calculated by dividing the mass or moles of exchanged atomic oxygen by the mass or moles of the OCM employed, respectively.

The chemical looping concept is exemplified below for the water gas shift reaction (shown in eq. 1, with eq. 2 and 3 indicating the corresponding half-reactions), where a simple metal oxide, MO, is being used as OCM. Complex metal oxides such as perovskites ABO_3_ can also be used, although their oxygen capacity δ is often considerably smaller than that of simple metal oxides (e.g., δ_M_ typically between 0.05–0.1 but can reach up to 0.5 for certain perovskites)^5,11–13^.

1 $${\rm{CO}}+{{\rm{H}}}_{2}{\rm{O}}\to {{\rm{CO}}}_{2}+{{\rm{H}}}_{2}$$

2 $${\rm{CO}}+{\rm{MO}}\to {{\rm{CO}}}_{2}+{\rm{M}}$$

3 $${\rm{M}}+{{\rm{H}}}_{2}{\rm{O}}\to {\rm{MO}}+{{\rm{H}}}_{2}$$

Chemical looping also allows for safer operating conditions, removes the need for product separation and allows the immediate capture of resulting greenhouse gases such as CO_2_^9,14^. Most importantly, because mixing reactant gases is avoided by transferring oxygen from the oxidizing H_2_O stream to the reducing CO stream via the perovskite OCM, this allows for surpassing equilibrium limitations which are inherent to typical mixed reactions. In turn, this can potentially increase conversion and selectivity^15^. The OCM is often a simple metal/metal oxide (M/MO) which can undergo reversible redox reactions and display suitable thermodynamics for the desired application. For example, for the water gas shift reaction, a Fe/FeO_x_ system (where x = 1, 1.33 and 1.5 for FeO, Fe_3_O_4_ and Fe_2_O_3_, respectively) is often used^9^. One of the limitations of this system (and that of other composites in general) is that it can easily agglomerate over time^16–18^. Recently, it has been suggested that agglomeration may be avoided by encapsulating the oxygen storage material Fe/FeO_x_ in a more redox resilient, yet fast oxygen transport material, such as a perovskite, La_1−x_Sr_x_FeO_3_, resulting in a complex composite material^19^. Preliminary work has indicated that this could be used for multiple (i.e. 25) cycles for hydrogen production via the water-gas shift reaction with little decrease in performance^19^. While these initial results are encouraging, durability should be investigated over a larger number of cycles to probe the generality and larger scale applicability of this approach. Moreover, it is worth noting that in chemical looping, as well as in the other energy conversion technologies mentioned above, the effect of redox cycling on performance and durability is often only inferred from macroscopic scale techniques that average physico-chemical properties over a large number of composite particles. Little attention has been paid so far to the processes occurring at the individual particle level, which must underpin the overall macroscopic behaviour.

Here, we follow the performance and microstructure of a complex metal-perovskite oxide composite material for packed-bed chemical-looping production of hydrogen over 200 cycles – much longer than previous studies on similar systems. We isolate several individual particles of a few tens of microns in diameter and track their 3D microstructural evolution during cycling by means of multi-length scale X-ray computed tomography (X-ray CT) to understand the microstructural evolution of the composite in relation to its activity. Unlike previous studies, we investigate the correlation between microstructural evolution at the particle level and H_2_ production at the powder level. We show that upon redox cycling the particle develops a large amount of internal porosity, which initially boosts activity, but on the long term facilitates structural and compositional reorganisation and eventually degradation. We also study the composite by *in situ* XRD, during one redox cycle, to provide initial information on phase evolution upon exposure to redox conditions. Overall, our results provide interesting new insight into the driving forces behind reorganization at the particle composite level in relation to reactivity, providing insight into various options for controlling it.

### Composite systems and their activity evolution on redox cycling

**Figure 1:**
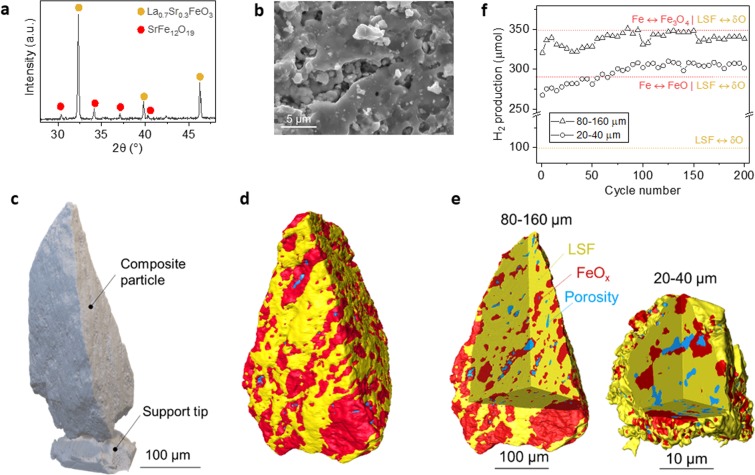
Composite systems and their activity evolution during redox cycling. (**a**) Room temperature XRD scan and phase identification. (**b**) SEM micrograph of the composite surface. (**c**) 3D reconstruction from X-ray CT data of a 80–160 µm composite particle glued on the tip of a needle. (**d**) Phase distribution and microstructure of the composite particle shown in (**c**) derived by X-ray CT analysis. Phases denoted by red and yellow colours corresponds to (**a**) while blue denotes porosity. (**e**) 3D reconstruction of as-prepared composite particles of different size carried out by X-ray CT. (**f**) H_2_ production by chemical looping from a packed-bed reactor at 850 °C, using composite particles of size exemplified in (**e**). The plot also highlights contribution to H_2_ production from the LSF matrix as well theoretical limits when the Fe metal phase is converted entirely to FeO or Fe_3_O_4_.

The composite system employed in this study comprises 30 wt.% Fe_2_O_3_ within a La_0.7_Sr_0.3_FeO_3_ (LSF) perovskite oxide matrix. The loading was selected to reduce the likelihood of interaction between the iron particles, which could lead to sintering^19^. In the as-prepared form, analysis of X-ray diffraction (XRD) patterns confirmed the presence of a majority perovskite phase, La_0.7_Sr_0.3_FeO_3_, while the Fe_2_O_3_ was incorporated as strontium hexaferrite, SrFe_12_O_19_ (Fig. 1a). Scanning electron microscopy (SEM) revealed the composite particles display a rather rough and porous surface microstructure (Fig. 1b). In order to better understand the internal microstructure of the composite and the phase distribution within it, individual composite grains were isolated from the as-prepared system (in powder form), glued onto a needle (Fig. 1c) and analysed by X-ray CT. This revealed that the SrFe_12_O_19_ particles are generally micron size and uniformly embedded within the perovskite oxide matrix, with a small amount of porosity being present (Fig. 1d).

To investigate the impact of the size of the composite particles on the redox durability and activity, the system was prepared in two different particle size ranges, small (20–40 µm) and large (80–160 µm), Fig. 1e. Individual particles from these samples were then selected and tracked during redox cycling. We initially attempted to investigate multiple particles of each size, however, manipulating such fine particles and transferring them between the testing and characterization equipment proved to be extremely challenging. Due to these reasons, only one particle of each size was employed throughout the experimental procedure. However, as will be shown, both display similar microstructural and performance behavior and evolution, implying that they can be considered as being representative for the studied system. Figure 1f shows the H_2_ production in micromoles for the two systems with different particle size, over 200 isothermal redox cycles at 850 °C. It should be noted that in the case of this composite, both the Fe/FeO_x_ phase and the LSF phase are expected to contribute to H_2_ production, as shown previously^19^, with phase contributions highlighted in Fig. 1f.

Both composites show oxygen capacity values and corresponding H_2_ production values characteristic of LSF and LSF-based composites generally reported in the literature^5,19^. For example, the LSF perovskite alone displays a H_2_ production of ~100 µmol H_2_ (for 50 mg sample, see experimental section), resulting in a δ_m_ of ~3.2%, consistent with previous reports^5,19^. The composite with smaller particle size exhibited H_2_ production values from 275 to 300 µmol H_2_, corresponding to δ_m_ of 8.8 and 9.6%, respectively. The composite with larger particle size exhibited slightly higher H_2_ production, ranging from 325 to 350 µmol H_2_, corresponding to δ_m_ of 10.4 and 11.2%, respectively. As mentioned above, these are in line with oxygen capacity values generally reported in the literature for such composites^4,5,9^.

The maximum H_2_ production for the two systems seems to coincide with the average oxidation state iron can reach, which seems to be close to Fe_3_O_4_ for the large particle system and FeO for the small particle system. Based on the current data it is difficult to determine the exact cause of this effect but is could be due to the fact that as the particle size decreases, the perovskite matrix might be able to contain or restrict to a higher extent the volume expansion associated with the oxidation of Fe to FeO_x_, only allowing oxidation to FeO. Interestingly, however, both composites displayed an initial increase in hydrogen production during redox cycling, followed by a plateau region. The sample with larger particles also displayed a slight activity decline after about 150 cycles (Fig. 1f).

### Phase evolution during redox cycling

**Figure 2:**
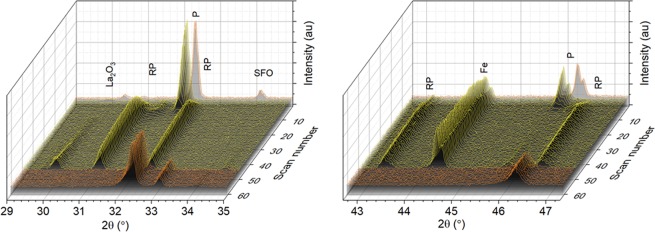
Composite phase analysis by *in situ* XRD during one redox cycle. The experiment was carried out at 850 °C. The sample was initially held in air for one 60 min (scans 1,2), then the environment was switched to H_2_ for 650 min (scans 3 to 52), and then back to air for an additional 800 min (scans 53 to 64). The time elapsed to scan from 25° to 50° 2-theta was varied between 7 and 13 minutes (see the experimental section for details).

In order to obtain information on the phase changes occurring in the composite during the initial exposure to redox conditions, we carried out *in situ* XRD over one redox cycle, using the sample with large particles. Due to limitations of the *in situ* XRD setup, for this experiment 5% H_2_ was used during the reduction cycle and air was used for the oxidation cycle, rather than CO and steam, respectively. The phases identified at room temperature (RT) remained stable under air up to 850 °C, where they were held for one hour (scans 1, 2 in Fig. 2). The environment was then switched to H_2_ for 650 minutes (scans 3 to 52 in Fig. 2), and then back to air for an additional 800 minutes (scans 53 to 64, Fig. 2). The time elapsed to scan from 25° to 50° 2-theta was varied between 7 and 13 minutes (see the experimental section for details).

Immediately upon the introduction of H_2_, the perovskite peaks shift to lower two-theta angles, which is indicative of perovskite reduction, while the SrFe_12_O_19_ peaks disappear and metallic Fe (α-phase) appears. This indicates that the reduction of the perovskite and SrFe_12_O_19_ phase occur very fast (in a matter of minutes) and these processes are probably linked since the iron oxide phase is embedded in the perovskite and the latter will mediate oxygen transport. Although the perovskite peaks persist for over 100 minutes under H_2_ (scans 3 to 13), their intensity decreases gradually, while, at the same time, a Ruddlesden-Popper phase, La_y_Sr_2−y_FeO_4_, develops soon after the start of hydrogen flow (scan 6). Shortly after, La_2_O_3_ also starts to appear (scan 18) and towards the end of the reduction (scan 37), SrO also appears in low concentrations. As compared to the initial perovskite, the La_y_Sr_2−y_FeO_4_ phase appeared to be more kinetically stable in the H_2_ environment, surviving the full length of the reduction, with a slight decrease in peak intensities observed with a simultaneous minor increase in the iron, lanthanum oxide, and strontium oxide peak intensities.

In contrast to the reduction, the re-oxidation of the composite occurred almost immediately, with all phase evolution being completed by the first 7 minute scan (scan 53 in Fig. 2). The peaks of the re-oxidised composite were found to correspond to the LSF perovskite phase as well as hematite Fe_2_O_3_ and a small amount of strontium SrFe_12_O_19_. This indicates that oxidation did not revert the composite to its original state, hence the phase composition is not entirely redox reversible. It should be also noted that the perovskite peaks of the oxidised phase are slightly broader and less intense than after the cycling, suggesting that the average crystallite sizes for the perovskite phase decreases upon re-oxidation, consistent with X-ray CT described later. This indicates that the redox process fragments the perovskite oxide matrix to some extent, leading to the creation of defects that on one hand could facilitate oxygen exchange, and on another hand facilitate subsequent restructuring. Overall, this implies that the redox cycle was not entirely reversible, both from a phase and a morphological point of view which suggests that the composite is prone to have a dynamic phase and microstructure, varying from cycle to cycle.

However, since the above XRD data was collected in slightly harsher redox conditions (and for longer half-cycle duration) than those employed for typical water gas shift experiment, the phase changes outlined above are expected to be more representative of a long-term operation behavior.

### Microstructural evolution during redox cycling

**Figure 3:**
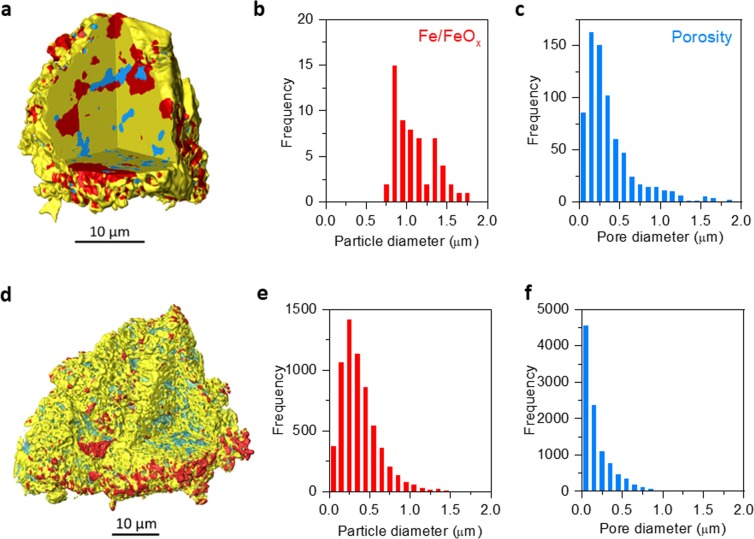
Microstructural evolution of a small composite particle after 200 redox cycles. Microstructures and particle size and pore analysis obtained for a small composite particle: (**a–c**) before and (**d**–**f**) after the 200 redox cycles shown in Fig. 1f.

Individual particles were analysed by X-ray CT to obtain a better understanding of the microstructure of the samples at different points during the cycling and in relation to their activity (Fig. 1f). Analysis of a single small particle before and after 200 redox cycles showed that the Fe-phase becomes highly fragmented and a large number of small pores develop (Fig. 3). Initially, the composite particle contains a few tens of FeO_x_ particles with an average size of 1–1.5 µm (Fig. 3a,b). After redox cycling, these particles re-disperse into hundreds of particles with an average size of 0.25 µm (Fig. 3d,e). A very similar trend is observed for the pore phase, where initially a few tens of 0.25 µm pores are present (Fig. 3c), evolving to thousands of pores of <0.1 µm average size (Fig. 3f). Overall, the fragmentation of the Fe-phase coupled with an increase in porosity means that the accessibility of the Fe-phase to the gas environment increases with redox cycling, accounting for the gradual increase in H_2_ production over the first 100 cycles (Fig. 1f).

**Figure 4:**
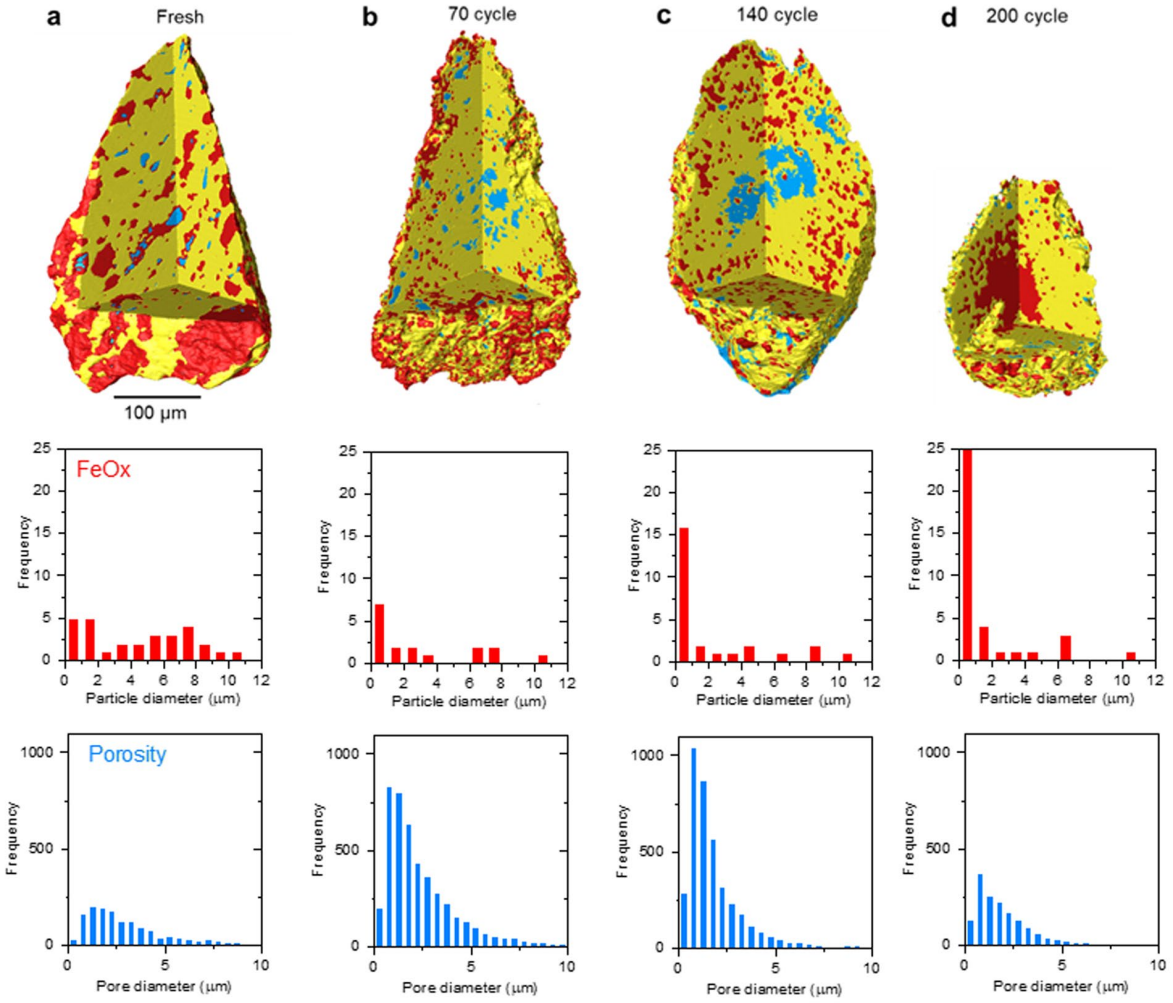
Microstructural evolution of a single, large composite particle during 200 redox cycles. Microstructure and Fe/FeO_x_ particle size and porosity analysis by X-ray CT for a single composite particle after (**a**) 0, (**b**) 70, (**c**) 140 and (**d**) 200 redox cycles. The particle was part of the packed-bed that displayed the H_2_ production activity plotted in Fig. 1f.

We tracked the evolution of a single large particle after 70, 140 and 200 redox cycles (Fig. 4) to get a better understanding of the different stages leading up to the reorganisation observed above. Quantitative CT analysis revealed a similar evolution as highlighted above, marked by a dramatic fragmentation of the Fe-phase as a well as that of the perovskite matrix as reflected by an increase in porosity. Again, this corroborates well with the observed increase in performance over the initial 50–100 redox cycles (Fig. 1f).

**Figure 5:**
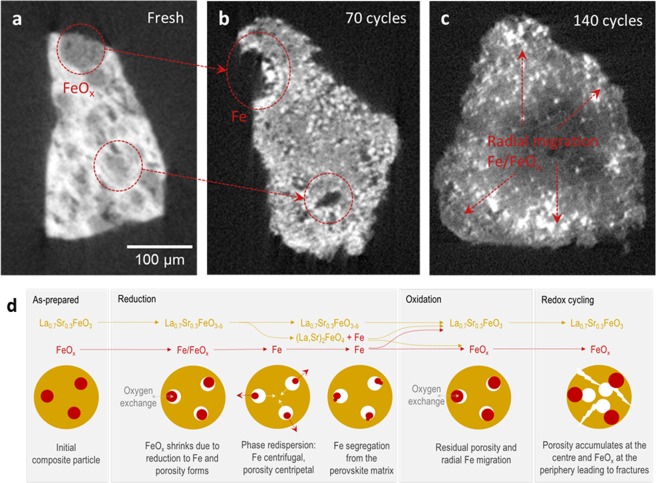
Porosity formation and Fe phase redispersion on redox cycling. Slices through the microstructures shown in Fig. 5 by X-ray CT, showing phase distribution (**a**) initially and (**b**,**c**) after 70 and 140 redox cycles, respectively. (**d**) Schematic diagram of the evolution of composite particles during redox cycling.

Close inspection of the X-ray CT data in Fig. 4 reveals that the redistribution of the Fe-phase and of the porosity are not random. Starting at 70 cycles (Fig. 4b), the Fe particles migrate towards the periphery of the particle, that is, in a centrifugal pattern with respect to a virtual centre of the particle, while the porosity accumulates in the middle of the particle. This trend becomes even more apparent after 140 cycles (Fig. 4c). Due to the increased Fe-phase concentration towards the periphery of the particle, this phase starts to agglomerate and sinter (see Fig. 5). This is also marked by a slight decrease in H_2_ production probably indicating a drop in the accessibility of the overall oxygen capacity. At the same time, the increased porosity at the middle of the particle seems to render it fragile. Indeed, after 200 cycles, the particle ruptured and the remaining FeO_x_ phase within seems to have coalesced considerably (Fig. 4d).

Overall, the X-ray CT data analysed above accounts well for the trend observed in the activity of the composites (Fig. 1f). Initially, there is a dramatic increase in porosity and fragmentation of the Fe-phase which probably increases oxygen exchange surface area, enabling better gas phase access to the Fe-phase that is more deeply embedded in the composite particle and thus boosting H_2_ production activity. However, at the same time, the large degree of fragmentation and porosity forming within the composite particle seems to facilitate further structural and compositional reorganisation and therefore degradation. This seems to correspond to the plateau region of H_2_ production activity where the increased activity due to increased porosity and Fe-phase redispersion is counterbalanced by deactivation due to incipient agglomeration and sintering. Once the sintering of the Fe-phase at the periphery of the particle starts to dominate in parallel with the clustering of porosity at the centre of the particle, this leads to an overall decrease in the accessibility of the material oxygen capacity, which is marked by a slight decrease in H_2_ production.

Finally, it is worth highlighting that the X-ray CT vividly illustrates the dynamic reshaping of the particle as it goes through subsequent redox cycles. This is consistent with the XRD analysis which pointed towards a phase and crystallite evolution of the particles and is also consistent with the somewhat fluctuating H_2_ production activity observed. Interestingly, after 70 cycles, the overall shape of the particle is still very similar to the initial one (compare Fig. 4a,b), but the shape changes considerably by 140 cycles (Fig. 4c), eventually disintegrating after 200 cycles (Fig. 4d) due to uneven phase redispersion, as discussed above.

### Mechanistic insight into the redox cycling evolution of a composite particle

The following mechanistic steps can be identified for the redox evolution of the composite particle (Fig. 5d) by combining the phase and microstructural information derived from XRD and X-ray CT. Initially, the composite consists of micron size iron oxide particles embedded in a perovskite oxide matrix with relatively low porosity. Upon reduction, the perovskite crystal structure expands (shift to lower 2θ in XRD, Fig. 2) while the iron oxide phase, on the contrary, shrinks drastically (~70% by volume) due to the transformation from Fe oxide to Fe metal. This leads to the formation of a large number of small pores within the perovskite matrix and possibly assists with crack formation or propagation (Fig. 5).

The XRD analysis indicates the reduction of the iron oxide phase occurs suddenly, which correlates well with the fact that X-ray CT shows the presence of a small, insular Fe metal phase along the surface of the perovskite matrix (Fig. 5b), as if the reduction occurred simultaneously and suddenly at different points at the embedded iron oxide particles. Where the edge of the composite particle and thus, communicating directly with the gas phase, their reduction and subsequent volume collapse leads to the formation of ‘bays’ decorated with Fe metal particles (Fig. 5b). Where the Fe oxide particles are embedded into the perovskite, their reduction will have to be mediated by the oxygen transport through the perovskite matrix. Since oxygen transport will normally occur via the shortest distance to the surface, the Fe-phase will naturally tend to relocate as close as possible to the surface of the composite particle where oxygen exchange occurs (highlighted in Fig. 5d), thus slowly migrating towards the periphery of the composite particle, in a centrifugal pattern, as observed above by X-ray CT. As illustrated in Fig. 5d, this will essentially cause the pores to gradually migrate towards the centre of the composite particle, in a centripetal pattern. However, the repeated volume changes are likely to contribute to migration due to the fact that during reduction, the FeOx particles within the composite can collapse into various arrangements of Fe islands and then re-aggregate upon oxidation, again in slightly probably in different configuration/morphology. This variability could very well contribute to Fe migration. The mass transport throughout the particle is likely facilitated by the compositional instability of the LSF matrix under redox conditions. As shown by XRD, LSF can potentially decompose under reducing conditions to form Fe metal and La_y_Sr_2−y_FeO_4_. Notably, porosity formation has been observed before to form under redox cycling of composites but to the best of our knowledge, the pattern in which this occurs has not been identified^20^.

Since oxidation will start from the composite particle surface this will further favour radial Fe-phase migration over time, while pushing pores towards the core of the particle. Additionally, since the Fe metal particles are now smaller than they were initially, they will oxidise faster, which is consistent with the results from XRD performed here. Thus, upon oxidation and volume expansion, this may lead to ‘asymmetric’ filling of the pores in which they used to reside. This, in turn will cause additional porosity formation and strain the perovskite, possibly creating cracks (Fig. 5d).

Last but not least, it is interesting to note that the collapse of porosity at the centre of the particle and the segregation of the metal at the periphery is strikingly reminiscent of the Kirkendall effect where metallic nanoparticles restructure into hollow-core nanoparticles upon oxidation due to vastly different diffusion rates of diffusion-coupled species (e.g. cations-anions or their respective vacancies)^21^. While the Kirkendall effect is usually observed at the nanoscale, here we observe a similar phase redistribution pattern over tens of microns.

## Conclusions

In this study we have demonstrated the use of X-ray CT for following the 3D transformation and internal phase redistribution of individual composite particles of tens of microns diameter during redox cycling. Our results also vividly illustrate that the morphological evolution of the composite particles during redox cycling is underpinned by a centrifugal redispersion of the embedded Fe phase and a centripetal clustering of the porosity (Fig. 5d) which is in turn driven by oxygen exchange via the perovskite matrix. Therefore, improving the performance and durability of such composites under redox conditions is intimately linked to the choice of materials and microstructure. Ideally, the perovskite oxide matrix would have to be compositionally more stable and morphologically more resilient (i.e. display less chemical expansion)^22^ under redox conditions. As shown above, the reversible redox decomposition of the LSF matrix to Fe metal and the Ruddlesden-Popper phase (prone to cleaving and favoring crack propagation) provides the means for Fe phase and porosity to redisperse within the composite particle.

Last but not least, we have shown how phase and microstructural changes at the particle level correlate and account for macroscopic properties such as oxygen exchange and H_2_ production over time, which are essential for understanding and developing more active and robust composite materials for application in energy conversion technologies.

## Methods

### Sample preparation

The composites were prepared according to the Pechini method described in detail previously^19^.

### Phase characterization from XRD

Lab X-ray diffraction (XRD) was performed in Bragg-Brentano geometry with a PANalytical Empyrean diffractometer using Cu Kα radiation (λ = 1.5406 Å). *In situ* measurements above room temperature were performed using an Anton-Paar XRK 900 furnace, with gas flow from either a gas cylinder (e.g. 5% H_2_ in N_2_) or a diaphragm pump to deliver air. Scans 1–47, 51, and 65–67 were recorded over 13 minutes from 25° to 50° 2-theta. Scans 52–61 were recorded over 7 minutes from 25° to 50° 2-theta to capture quicker processes. Scans 48–50 and 62–64 were recorded over 20 minute from 10°-to 90° 2-theta. Further scans in air upon re-oxidation are not shown, as no changes in XRD patterns were seen.

### X-Ray computed tomography

The samples with smaller particle size were analysed on the Zeiss Xradia 810 Ultra X-ray computed tomography microscope (Electrochemical Innovation Lab, University College London). In preparation for X-ray CT, particles were glued on the tip of a needle via two component epoxy resin. The mounted particles were then imaged using the large field of view in Zernike phase-contrast mode. Exposure time for the fresh and cycled sample amounted to 90 s and 60 s, respectively while the voxel size for both samples amounted to approximately 0.063 μm in each dimension using a camera binning of 1. For each sample, 901 projections were collected and reconstructed via a filtered back projection algorithm (Zeiss XRM Reconstructor).

The sample with larger particle size was analysed using the Zeiss Xradia 520 Versa X-ray microscope (Electrochemical Innovation Laboratory, University College London). The mounted particle (as mentioned above, on the tip of a needle) was then imaged using the specifications presented in Table 1. The scanning parameters were varied according to the sample structure and density to provide the best imaging signal-to-noise ratio. Each scan was then reconstructed via a filtered back projection algorithm (Zeiss XRM Reconstructor).

**Table 1:** X-ray micro CT imaging specifications.

		Fresh sample 70 cycles sample	140 cycles sample	200 cycles sample
Source voltage	[kV]	160		
Source power	[W]	10		
Objective	[×]	20	40	20
Camera binning	[-]	1	2	1
Voxel size	[μm]	0.383	0.42	0.37
Exposure time	[s]	47	11	30
Number of images	[-]	1851	3201	2001

The segmentation was conducted using gray-scale thresholding method based on the reconstructed CT data using Avizo 9.4 (Thermo Fisher Scientific, UK). Due to the connectivity and morphology of the microstructure, continuous particle/pore size distribution (C-PSD) developed by Holzer *et al*.^23^ was used for the quantification in open source software Fiji.

### Redox cycling and H_2_ production

Redox cycling was carried out based on a procedure and using an experimental setup reported previously^19^. For testing, a programmable, fully automated microreactor was used (CATLAB, Hiden Analytical, UK). Testing was carried out at 1 atm and 850 °C and for each test, 50 mg of sample were loaded in the microreactor. After this, the microreactor was purged with helium at 50 mL min^−1^ before commencing testing. The reduction cycle was carried out in 5% CO in helium, while the oxidation in 5% H_2_O in He, both with flow rates of 50 mL min^-1^ and duration of 30 min. After each reduction and oxidation step, the reactor was flushed with helium for 15 min, to evacuate any remaining oxidizing or reducing gases to prevent their mixing. The gases at the outlet of the microreactor, i.e. CO, CO_2_, H_2_, H2O and He were continuously analyzed by connecting the microreactor to a soft ionisation quadrupole mass spectrometer (QMS) via a heated capillary line. Sample temperature was measured and controlled by an internal K-type thermocouple secured into an alumina sheathing. A saturator system (Grant, UK) was used to deliver water into the microreactor, using helium as carrier gas. The water bath was set at 30 °C which, at atmospheric pressure, corresponds to a 4.2% mole fraction of water in the carrier gas. The amount of H_2_ produced was calculated by integrating the data consisting of produced hydrogen mole fraction as a function of time.

## Data Availability

The data supporting this publication is available at 10.25405/data.ncl.9849803.

## References

[1] Irvine JTS (2016). Evolution of the electrochemical interface in high-temperature fuel cells and electrolysers. Nature Energy.

[2] Inoishi A, Ida S, Uratani S, Okano T, Ishihara T (2012). High capacity of an Fe–air rechargeable battery using LaGaO3-based oxide ion conductor as an electrolyte. Phys. Chem. Chem. Phys..

[3] Xu N, Li X, Zhao X, Goodenough JB, Huang K (2011). A novel solid oxide redox flow battery for grid energy storage. Energy Environ. Sci..

[4] Zeng L, Cheng Z, Fan JA, Fan L-S, Gong J (2018). Metal oxide redox chemistry for chemical looping processes. *Nature Reviews*. Chemistry.

[5] Zhu X, Li K, Neal L, Li F (2018). Perovskites as Geo-inspired Oxygen Storage Materials for Chemical Looping and Three-Way Catalysis: A Perspective. ACS Catalysis.

[6] Michalsky R, Botu V, Hargus CM, Peterson AA, Steinfeld A (2015). Design Principles for Metal Oxide Redox Materials for Solar-Driven Isothermal Fuel Production. *Advanced Energy*. Materials.

[7] Lyngfelt A, Leckner B, Mattisson T (2001). A fluidized-bed combustion process with inherent CO2 separation; application of chemical-looping combustion. Chemical Engineering Science.

[8] Fan, L.-S. *Chemical looping systems for fossil energy conversions*. (Wiley-AIChE, 2010).

[9] Thursfield A, Murugan A, Franca R, Metcalfe IS (2012). Chemical looping and oxygen permeable ceramic membranes for hydrogen production – a review. Energy & Environmental Science.

[10] Kathe MV, Empfield A, Na J, Blair E, Fan L-S (2016). Hydrogen production from natural gas using an iron-based chemical looping technology: Thermodynamic simulations and process system analysis. Applied Energy.

[11] Rydén M (2008). Novel oxygen-carrier materials for chemical-looping combustion and chemical-looping reforming; LaxSr1−xFeyCo1−yO3−δ perovskites and mixed-metal oxides of NiO, Fe2O3 and Mn3O4. International Journal of Greenhouse Gas Control.

[12] Albrecht KJ, Jackson GS, Braun RJ (2016). Thermodynamically consistent modeling of redox-stable perovskite oxides for thermochemical energy conversion and storage. Applied Energy.

[13] Marek E, Hu W, Gaultois M, Grey CP, Scott SA (2018). The use of strontium ferrite in chemical looping systems. Applied Energy.

[14] Qin L (2017). Improved cyclic redox reactivity of lanthanum modified iron-based oxygen carriers in carbon monoxide chemical looping combustion. Journal of Materials Chemistry A.

[15] Metcalfe, I. S. *et al*. Overcoming chemical equilibrium limitations using a thermodynamically reversible chemical reactor. *Nature Chemistry*, 10.1038/s41557-019-0273-2. 10.1038/s41557-019-0273-2 31133740

[16] Brown TA, Scala F, Scott SA, Dennis JS, Salatino P (2012). The attrition behaviour of oxygen-carriers under inert and reacting conditions. Chemical Engineering Science.

[17] Imtiaz Q, Kurlov A, Rupp JLM, Müller CR (2015). Highly Efficient Oxygen-Storage Material with Intrinsic Coke Resistance for Chemical Looping Combustion-Based CO2 Capture. ChemSusChem.

[18] De Vos Y (2019). Sustainable iron-based oxygen carriers for Chemical Looping for Hydrogen Generation. International Journal of Hydrogen Energy.

[19] Dueso C, Thompson C, Metcalfe I (2015). High-stability, high-capacity oxygen carriers: Iron oxide-perovskite composite materials for hydrogen production by chemical looping. Applied Energy.

[20] Qin L, Majumder A, Fan JA, Kopechek D, Fan L-S (2014). Evolution of nanoscale morphology in single and binary metal oxide microparticles during reduction and oxidation processes. J. Mater. Chem. A.

[21] Yin Y (2004). Formation of Hollow Nanocrystals Through the Nanoscale Kirkendall Effect. Science.

[22] Bishop SR (2014). Chemical Expansion: Implications for Electrochemical Energy Storage and Conversion Devices. Annual Review of Materials Research.

[23] Münch B, Holzer L (2008). Contradicting Geometrical Concepts in Pore Size Analysis Attained with Electron Microscopy and Mercury Intrusion. Journal of the American Ceramic Society.

